# Characteristics of Acute Kidney Injury (AKI) in Children With Cancer and in the Bone Marrow Transplant Unit: An Analysis of an AKI Registry From a Tertiary Cancer Center

**DOI:** 10.7759/cureus.98875

**Published:** 2025-12-10

**Authors:** Edward J Saca, Hasan Hashem, Zebin Al Zebin, Tariq Mohammad, Omar Banat, Mohammad Salameh, Ezaldeen Azzeh, Mohammad Saleem, Rawad Rihani

**Affiliations:** 1 Department of Pediatric Nephrology, King Hussein Cancer Center, Amman, JOR; 2 Department of Pediatric Nephrology, Ibn Al Haytham Hospital, Amman, JOR; 3 Department of Pediatrics, King Hussein Cancer Center, Amman, JOR; 4 Department of Pediatrics, Ibn Al Haytham Hospital, Amman, JOR; 5 Department of Pediatric Critical Care, King Hussein Cancer Center, Amman, JOR; 6 Department of Pediatric Oncology, King Hussein Cancer Center, Amman, JOR

**Keywords:** acute kidney injury, bone marrow transplant, childhood cancer, critical care, nephrotoxic medications, renal replacement therapy, residual renal damage

## Abstract

Objective

Detection and tracing of acute kidney injury (AKI) in children with cancer in Jordan and the developing world are very limited. The objective of this paper is to report the incidence, the outcome, risk factors, as well as some clinical parameters associated with AKI in children with cancer.

Methodology

This study is based on an analysis of a registry that was established for children with cancer who develop AKI. The aim of the registry was to detect, trace, define risk factors, and maintain follow-up for children who develop AKI at King Hussein Cancer Center (KHCC). A flag system was established for all children who develop AKI. The pediatric department at KHCC is composed of four locations. These include the pediatric ward, bone marrow transplant (BMT) unit, pediatric intensive care unit (PICU), and the emergency room (E/R). Analysis was done in all locations. AKI was defined and staged according to the Kidney Disease: Improving Global Outcomes (KDIGO) criteria.

Results

During the study period, there were 16,273 total hospital admissions, and the cancer registry included 4,482 unique patients. A total of 473 AKI attacks occurred, representing 2.9% of all admissions. These attacks occurred in 374 patients, indicating that 8.3% of cancer patients experienced at least one AKI episode. The mean follow-up duration was 2.27 ± 1.28 years.

In the pediatric ward, 134 attacks of AKI were recorded (1.1% of admissions; 5.7% of patients). In the BMT unit, 117 attacks of AKI were recorded (4.3% of admissions; 6.9% of patients). In the PICU, 74 attacks of AKI were recorded (4.4% of admissions; 10.8% of patients). The most common place of developing AKI for patients at KHCC was in the home setting (community acquired) as 148 (31.3%) of patients who had AKI presented to the E/R with AKI, 134 (28.3%) had AKI while in the ward, 117 (24.7%) patients while in the BMT unit, and 74 (15.6%) patients while in the ICU. Fifty-four (14.4%) patients had two attacks, 15 (4.0%) had three attacks, and five (1.3%) had more than three attacks of AKI.

The maximum stage of AKI reached was stage 1 in 204 (43.1%) patients, stage 2 in 167 (35.3%) patients, and stage 3 in 102 (21.6%) patients. In the PICU stage, three was the most common maximum stage reached. The main cause of AKI at KHCC was nephrotoxic medications in 204 (43.1%) of patients. High-dose methotrexate (MTx) was the most common single drug to cause AKI in 34 (16.7%) patients. The most common cause for home-acquired AKI was pre-renal in 47 (32%) patients.

The most common malignancy in children who developed AKI was hematological in 230 (48.6%) patients. Residual renal damage was detected following 66 (14%) patients. Another 38 (8%) patients passed away, suggesting a higher mortality in children who developed AKI than in children without AKI. Renal replacement therapy (RRT) was done for 15 patients (3.2%), whereas 458 patients (96.8%) did not need RRT.

Conclusion

AKI in children with cancer shares many features with AKI in the general pediatric population, with multiple similar clinical manifestations. However, oncology patients have unique risk factors - most notably nephrotoxic chemotherapy, tumor lysis syndrome, sepsis, and obstructive uropathy - which distinguish their AKI profile. Variations between units within the same center can also influence AKI patterns. An in-center registry is therefore essential for early detection and ongoing monitoring of AKI cases.

## Introduction

Acute kidney injury (AKI) is a common condition affecting up to 30% of critically ill children in the ICU setting, as well as non-critically ill children admitted to pediatric wards [1-5]. It is a preventable and treatable disorder that occurs in multiple clinical settings inside the hospital or at home (community-acquired) [6,7]. Severity ranges from minor elevation of serum creatinine to a full-blown picture of acute renal failure [7]. The term AKI has rapidly replaced acute renal failure, as the latter is merely a description of the abrupt loss of kidney function that results in a decline in glomerular filtration rate (GFR) and, therefore, retention of nitrogenous waste products. The term AKI, on the other hand, and its stratified definition reflect that it is a disease entity with a possible continuum rather than an incidental condition [6]. It also provides a unified definition for study and research purposes.

The importance of detecting AKI, even in those with minor elevation of serum creatinine, is highlighted by the nature of life-threatening complications and the increased morbidity and mortality that may be associated with AKI, including fluid overload and hyperkalemia [7-9]. Several large single-center studies demonstrated that small changes in kidney function occur in more than a third of hospitalized patients and are associated with increased risk for mortality and morbidity [4,10,11].

Tracing of AKI remains deficient in our part of the world (developing countries) [7], especially in Jordan, where proper monitoring of AKI is almost nonexistent despite growing evidence that links AKI, especially recurrent episodes, to CKD [7,12]. Patients with AKI usually do not receive long-term follow-up by a nephrologist. Although management strategies for mild AKI are relatively simple, they may have a substantial impact on long-term renal outcomes. Pediatric patients have a long life expectancy, making it especially important for them to receive the highest standard of care.

## Materials and methods

At King Hussein Cancer Center (KHCC), we established a registry for AKI and a flag system for all children who develop AKI. The AKI flag system was activated based on a notification submitted by the assigned members of the AKI team to the Information Technology Department upon diagnosing a patient with AKI according to Kidney Disease: Improving Global Outcomes (KDIGO). Once activated, the system generated a flagged message visible to the relevant healthcare providers and remained attached to the patient’s electronic record for future reference. This study is based on the analysis of this registry. The aim of the registry was to detect and trace AKI cases, identify risk factors, and maintain follow-up for children who developed AKI at KHCC. AKI was defined and staged according to KDIGO criteria (Table 1) [13,14].

**Table 1:** KDIGO definition and staging of acute kidney injury KDIGO: Kidney Disease: Improving Global Outcomes; SCr: serum creatinine

Staging	Creatinine criteria	Urine output criteria
1	SCr rise ≥0.3 mg/dL within 48 hours or an increase in creatinine of ≥50% within seven days	>0.5 and ≤1 mL/kg/hr
2	Increase in creatinine of ≥100%	>0.3 and ≤0.5 mL/kg/hr
3	Increase in creatinine of ≥200% or SCr ≥4 mg/dL or receipt of dialysis or eGFR<35 mL/min/1.73 m^2^	≤0.3 mL/kg/hr

All children who developed AKI were registered in the registry. The registry contains all essential information, including demographics, gender, age, underlying disease, date of diagnosis, date of AKI development, treatment received, cause of AKI, as well as multiple risk factors associated with AKI development. Residual renal damage was defined as either failure of serum creatinine to return to baseline, a calculated estimated GFR (eGFR) using the original Schwartz formula of <90 mL/min/1.73 m^2^, persistent hypertension, or the presence of urinary sediment in the form of microscopic hematuria and non-nephrotic proteinuria. High-dose methotrexate (MTx) nephrotoxicity was defined as a dose greater than 500 mg/m^2^ [15].

Despite the availability of continuous renal replacement therapy (CRRT) at KHCC, currently considered the state-of-the-art RRT modality for AKI management, it is important to note that this modality is not yet well established in the pediatric ICU for technical reasons. The registry was established in January 2018, and all patients who developed AKI from that date onward were included. This paper represents an analysis of the registry over a period of nearly 4.5 years. Data from the registry were extracted into Microsoft Excel sheets (Microsoft Corp., Redmond, WA, USA) and subsequently exported to IBM SPSS Statistics for Windows, Version 25 (Released 2017; IBM Corp., Armonk, New York, United States) for statistical analysis.

## Results

**Figure 1:**
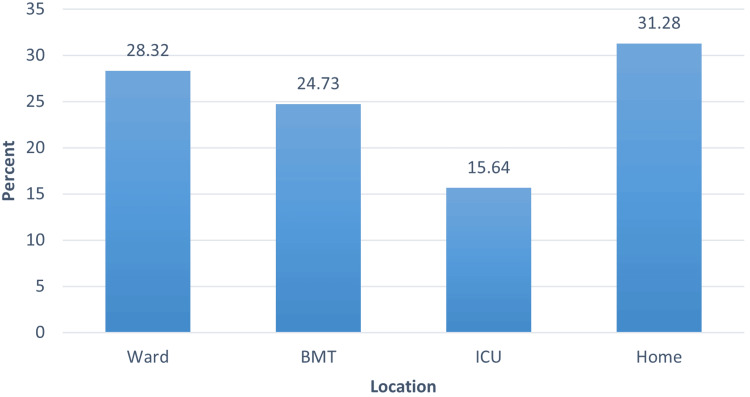
Percentage of AKI according to location AKI: acute kidney injury; BMT: bone marrow transplant; ICU: intensive care unit

The incidence of AKI at KHCC was 10.55% among patients, of whom 3.3% were community-acquired and 7.2% hospital-acquired. Excluding patients with community-acquired AKI, the incidence of AKI among patients at KHCC was 19.97 per 1000 admissions. Among those who developed AKI, 289 (61.1%) patients were males and 184 (38.9%) were females. The mean age at the time of AKI development was 11.33 ± 5.44 years. Males were slightly older than females, 11.54 ± 5.30 years as compared to 11.00 ± 5.66 years, but not statistically significant. Almost one-third of patients who had AKI, 148 (31.3%), acquired AKI while at home (Figure 1). It is notable that the most common setting for AKI development at KHCC, regardless of cause, was the home environment.

The incidence of AKI among pediatric ward patients was the lowest, at 11/1000 admissions, whereas in the bone marrow transplant (BMT) unit, an incidence of 43/1000 admissions was recorded, and in the ICU, where the incidence was 44/1000 admissions, the highest among all locations. The incidence was much higher, though, when calculated per patient rather than per admission, with an overall incidence of 10.55%. The incidence in the pediatric ward was 5.7%, in the ICU, it was 6.9%, whereas 10.8% of patients admitted to the BMT unit developed AKI.

**Figure 2:**
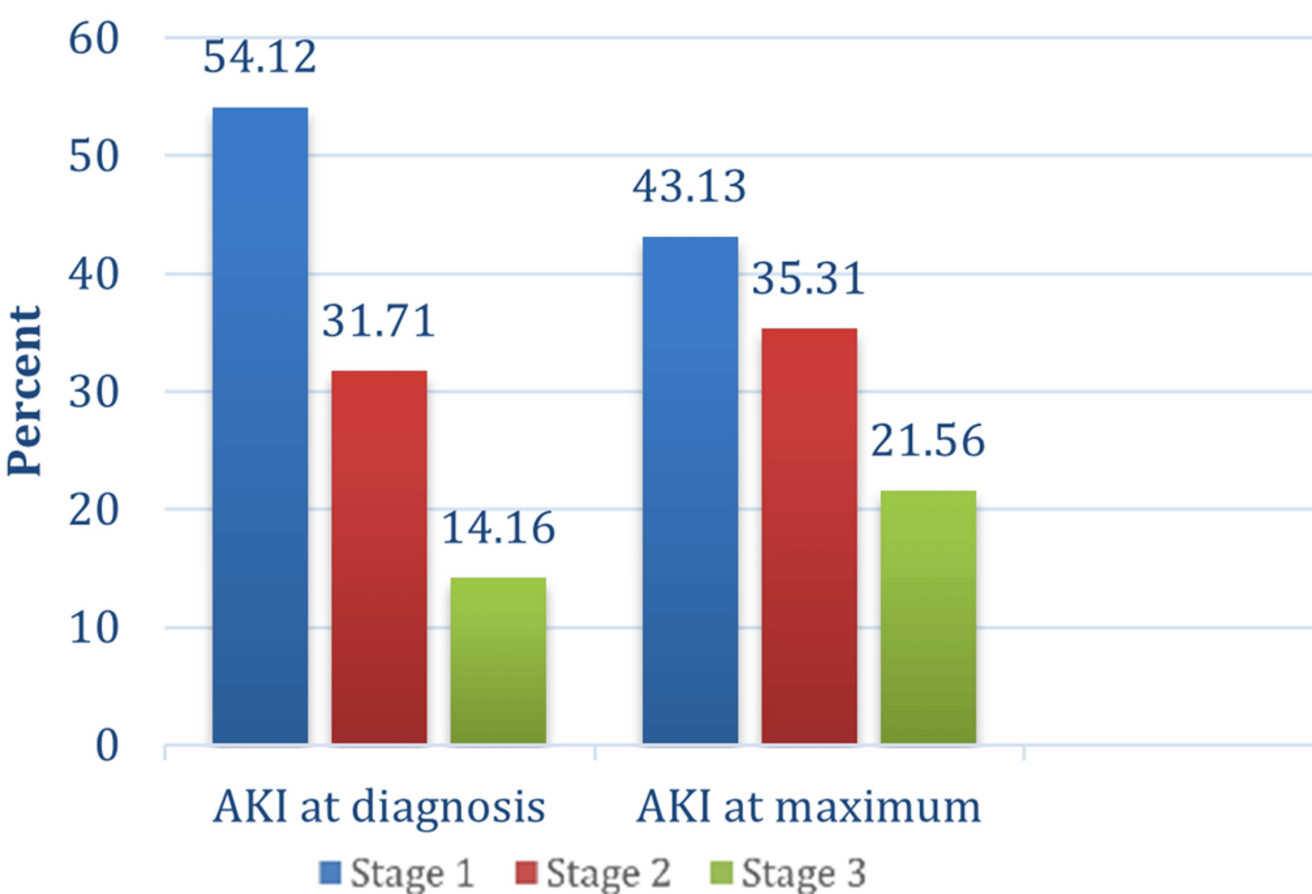
Stages of AKI at diagnosis and maximum stage reached AKI: acute kidney injury

Stage 1 AKI was the most common across all patients and locations, affecting 54.1% at AKI diagnosis and 43.1% at AKI maximum stage (Figure 2).

**Figure 3:**
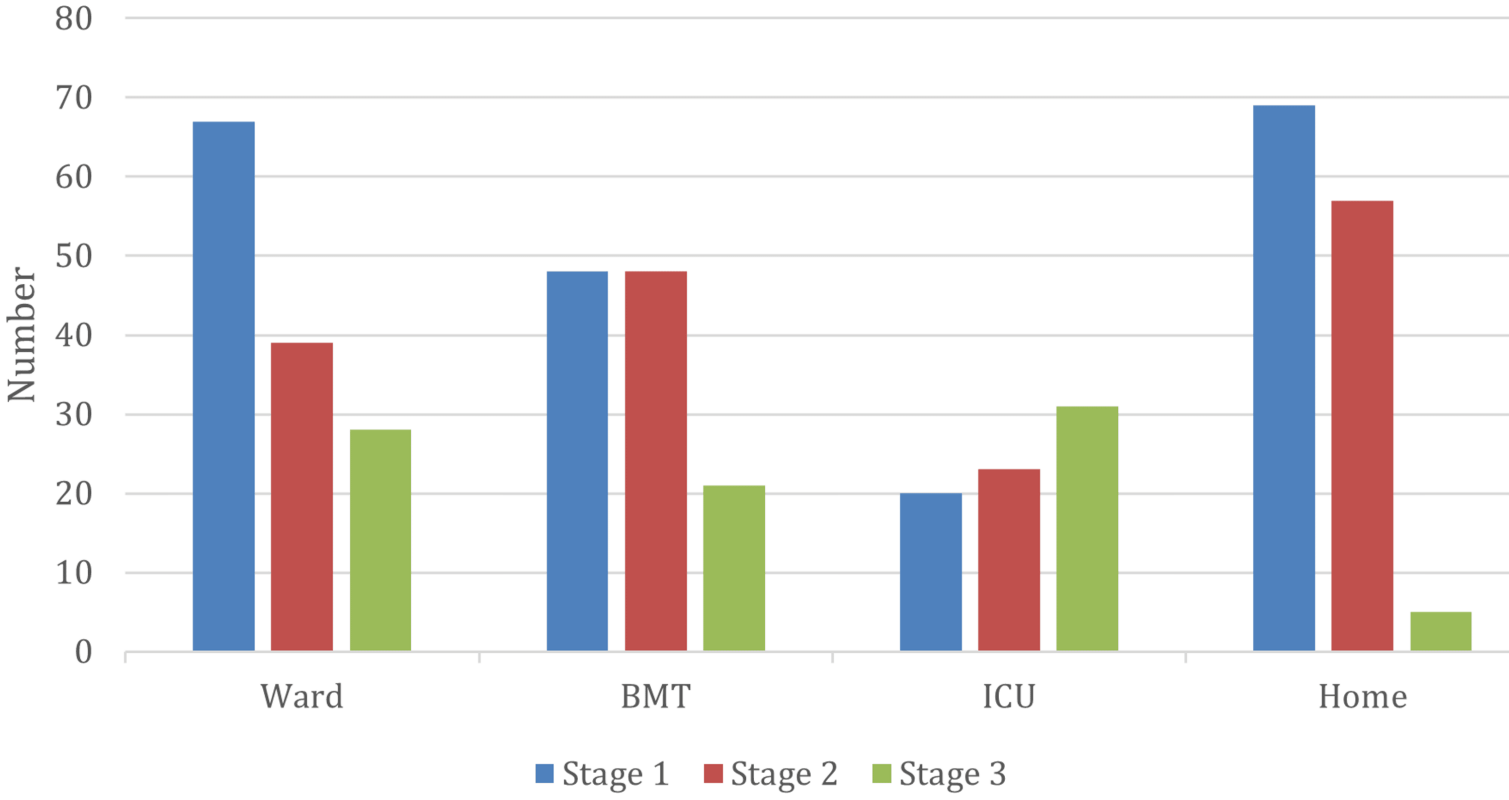
Maximum stage of AKI according to place AKI: acute kidney injury; BMT: bone marrow transplant; ICU: intensive care unit

The most common stage at diagnosis was stage 1 AKI in 256 (54.1%) patients, followed by stage 2 in 150 (31.7%) patients and stage 3 in 67 (14.2%) patients. The maximum AKI stage reached was stage 1 in 204 (43.1%) patients, stage 2 in 167 (35.3%) patients, and stage 3 in 102 (21.6%) patients. Stage 1 AKI was more common in all locations at diagnosis and as the maximum stage reached, except in the ICU, where stage 2 was the most common maximum stage reached (Figure 3). Overall, 52 (20.3%) patients progressed from stage 1 at diagnosis to stage 2 and stage 3 AKI. Seventeen (6.64%) patients progressed to stage 2, and 35 (13.67%) progressed to stage 3.

**Figure 4:**
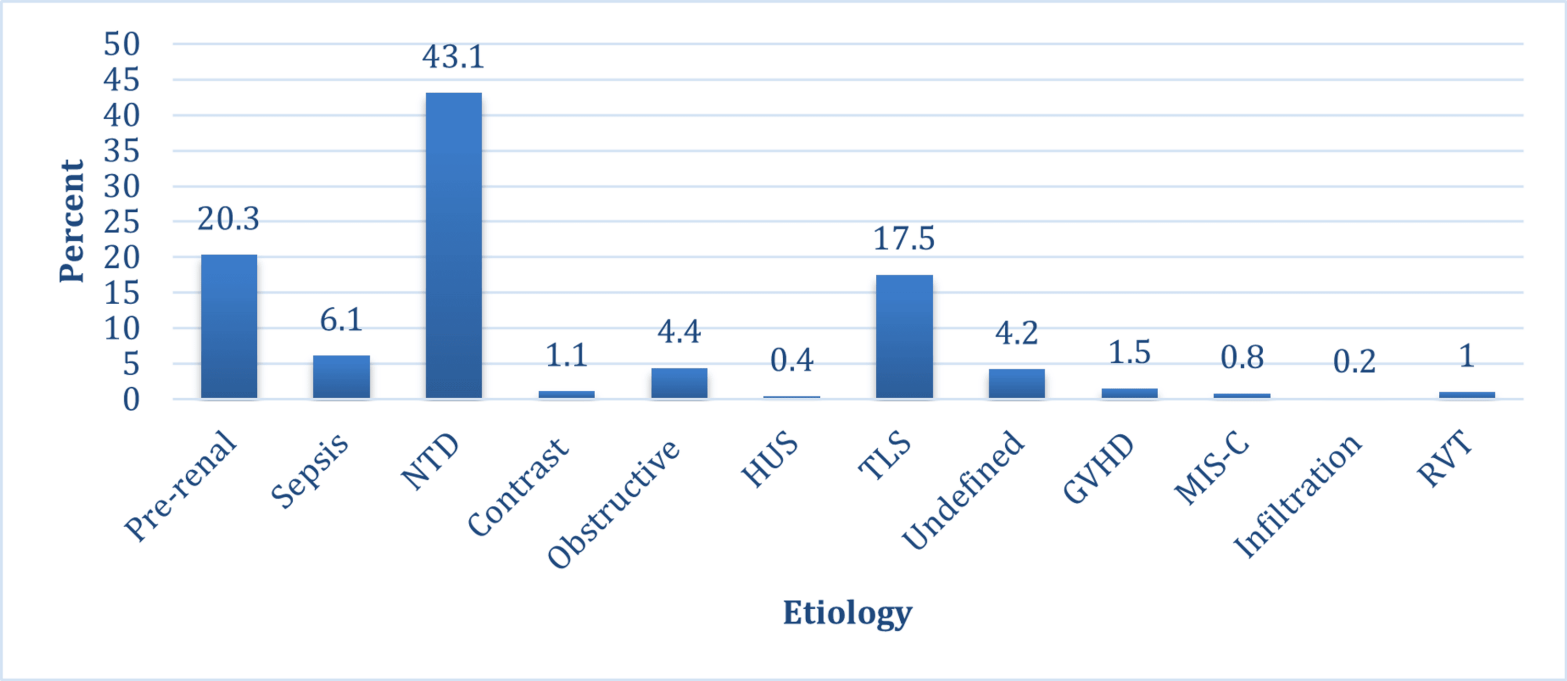
Etiology of AKI AKI: acute kidney injury; NTD: nephrotoxic drug; HUS: hemolytic uremic syndrome; TLS: tumor lysis syndrome; GVHD: graft-versus-host disease; MIS-C: multisystem inflammatory syndrome in children; RVT: renal vein thrombosis

The most common cause of AKI at KHCC was nephrotoxic medications, as it was implicated as the cause of AKI in 204 (43.1%) patients, followed by pre-renal AKI in 96 (20.3%) patients and tumor lysis syndrome (TLS) in 83 (17.5%) patients (Figure 4).

Nephrotoxic medications were the most common cause of AKI in patients who developed AKI in the ward and the BMT unit. In the ward, nephrotoxic drugs (NTDs) accounted for 60 (44.8%) cases, and in the BMT unit, NTDs accounted for a striking 93 (79.5%) AKI cases. The scenario was different in the ICU, where the most common cause of AKI was TLS, accounting for 21 (28.4%) cases, followed by sepsis in 14 (18.9%) cases and pre-renal AKI in 13 (17.6%) cases. In the ICU, nephrotoxic medications accounted for only 10 (13.5%) cases. In community- or home-acquired AKI, the most common cause was pre-renal AKI, accounting for 47 (31.8%) cases, followed by NTD in 41 (27.7%) cases (Table 2).

**Table 2:** Etiology versus location HUS: hemolytic uremic syndrome; TLS: tumor lysis syndrome; GVHD: graft-versus-host disease; MIS-C: multisystem inflammatory syndrome in children; RVT: renal vein thrombosis; BMT: bone marrow transplant; ICU: intensive care unit

	Ward	BMT	ICU	Home	Total
Pre-renal, N (%)	16 (11.9)	20 (17.1)	13 (17.6)	47 (31.8)	96 (20.3)
Sepsis, N (%)	5 (3.7)	2 (1.7)	14 (18.9)	8 (5.4)	29 (6.1)
Nephrotoxic Drugs, N (%)	60 (44.8)	93 (79.5)	10 (13.5)	41 (27.7)	204 (43.1)
Contrast Nephropathy, N (%)	0 (0.0)	0 (0.0)	4 (5.4)	1 (0.7)	5 (1.1)
Obstructive Uropathy, N (%)	10 (7.5)	1 (0.9)	2 (2.7)	8 (5.4)	21 (4.4)
HUS, N (%)	0 (0.0)	0 (0.0)	2 (2.7)	0 (0.0)	2 (0.4)
TLS, N (%)	35 (26.1)	0 (0.0)	21 (28.4)	27 (18.2)	83 (17.5)
Undefined, N (%)	4 (3.0)	1 (0.9)	3 (4.1)	12 (8.1)	20 (4.2)
Hepatorenal Syndrome/GVHD, N (%)	2 (1.5)	0 (0.0)	3 (4.1)	2 (1.4)	7 (1.5)
MIS-C/COVID-19, N (%)	1 (0.7)	0 (0.0)	2 (2.7)	1 (0.7)	4 (0.8)
Disease Infiltration, N (%)	1 (0.7)	0 (0.0)	0 (0.0)	0 (0.0)	1 (0.2)
RVT, N (%)	0 (0.0)	0 (0.0)	0 (0.0)	1 (0.7)	1 (0.2)
Total, N (%)	134 (100.0)	117 (100.0)	74 (100.0)	148 (100.0)	473 (100.0)

**Figure 5:**
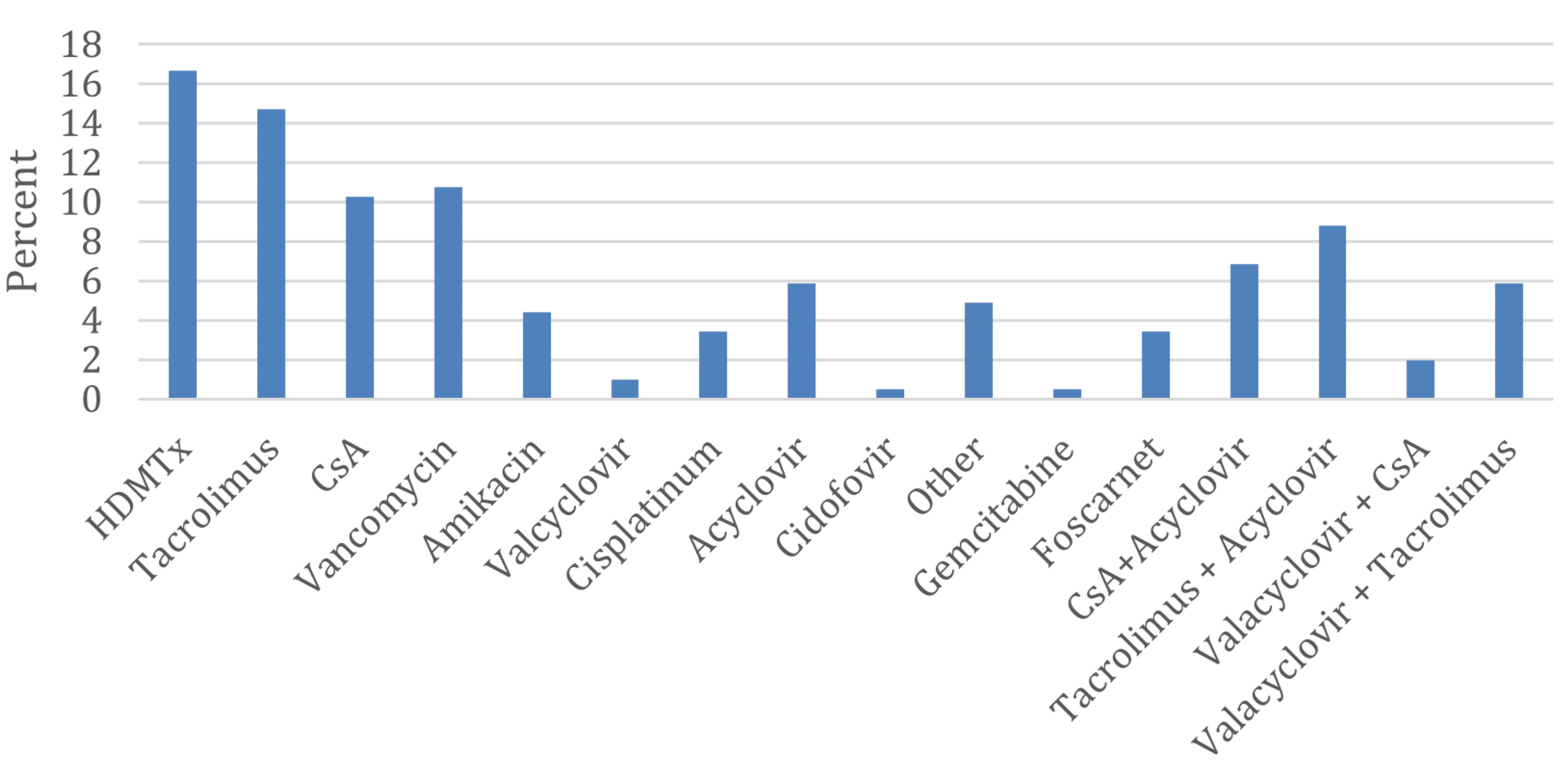
Nephrotoxic medications as a cause of AKI AKI: acute kidney injury; HDMTx: high-dose methotrexate; CsA: cyclosporine A

Most patients who developed AKI due to nephrotoxic medications were receiving more than one NTD. However, high-dose MTx was the most common single drug causing AKI, accounting for 34 (16.7%) cases, followed by tacrolimus in 30 (14.7%), vancomycin in 22 (10.8%), and cyclosporine A in 21 (10.3%). Notably, acyclovir and valacyclovir, either alone or in combination with a calcineurin inhibitor, were implicated in 62 (30.5%) cases of drug-induced AKI (Figure 5).

There were differences in the NTDs causing AKI across the different locations. In the ward, the most common drug was high-dose MTx, accounting for 29 (48%) of cases. In the BMT unit, calcineurin inhibitors alone accounted for 33 (35.5%) of cases, and when combined with acyclovir or valacyclovir, they accounted for 75 (80.6%) of cases.

In the ICU, vancomycin was the most frequent drug associated with AKI, occurring in four (40%) of cases, while in the home setting, tacrolimus predominated in 14 (34.1%) of cases (Table 3).

**Table 3:** Nephrotoxic medications according to location HDMTx: high-dose methotrexate; CsA: cyclosporine A; BMT: bone marrow transplant; ICU: intensive care unit

	Ward	BMT	ICU	Home	Total
HDMTx, N(%)	29(48.3)	0(0.0)	2(20.0)	3(7.3)	34(16.7)
Tacrolimus, N(%)	0(0.0)	16(17.2)	0(0.0)	14(34.1)	30(14.7)
CsA, N(%)	0(0.0)	17(18.3)	1(10.0)	3(7.3)	21(10.3)
Vancomycin, N(%)	15(25.0)	2(2.2)	4(40.0)	1(2.4)	22(10.8)
Amikacin, N(%)	6(10.0)	2(2.2)	0(0.0)	1(2.4)	9(4.4)
Valcyclovir, N(%)	0(0.0)	1(1.1)	0(0.0)	1(2.4)	2(1.0)
Cisplatinum, N(%)	3(5.0)	0(0.0)	0(0.0)	4(9.8)	7(3.4)
Acyclovir, N(%)	4(6.7)	4(4.3)	1(10.0)	3(7.3)	12(5.9)
Cidofovir, N(%)	0(0.0)	0(0.0)	1(10.0)	0(0.0)	1(0.5)
Other, N(%)	1(1.7)	2(2.2)	0(0.0)	6(14.6)	10(4.9)
Gemcitabine, N(%)	0(0.0)	0(0.0)	0(0.0)	1(2.4)	1(0.5)
Foscarnet, N(%)	0(0.0)	7(7.5)	0(0.0)	0(0.0)	7(3.4)
CsA + Acyclovir, N(%)	1(1.7)	11(11.8)	0(0.0)	2(4.9)	14(6.9)
Tacrolimus + Acyclovir, N(%)	1(1.7)	16(17.2)	0(0.0)	2(4.9)	19(8.8)
Valacyclovir + CsA, N(%)	0(0.0)	4(4.3)	0(0.0)	0(0.0)	4(2.0)
Valacyclovir + Tacrolimus, N(%)	0(0.0)	11(11.8)	0(0.0)	1(2.4)	12(5.9)
Total, N(%)	60(100.0)	93(100.0)	10(100.0)	41(100.0)	204(100.0)

**Figure 6:**
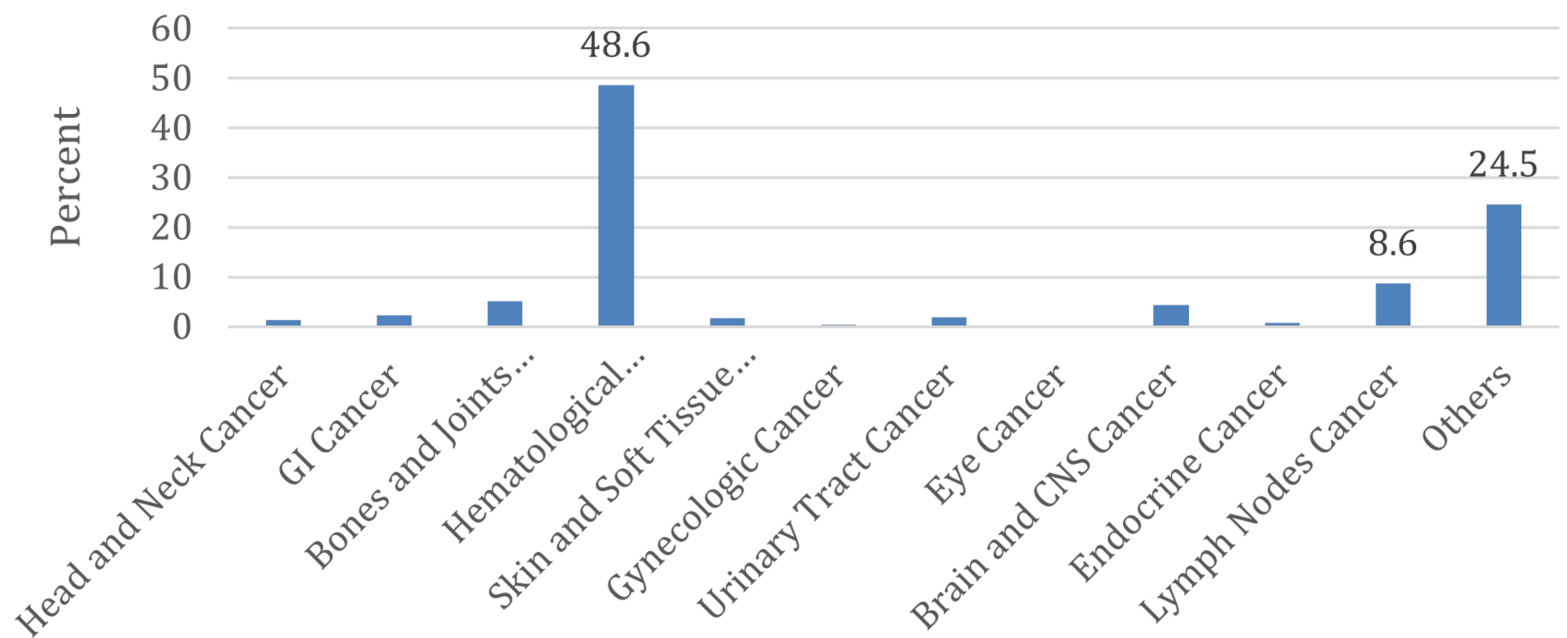
Types of malignancy

The most common malignancy in children who developed AKI was hematological malignancy, occurring in 230 (48.6%) patients, followed by lymphoma in 41 (8.7%) patients (Figure 6).

**Figure 7:**
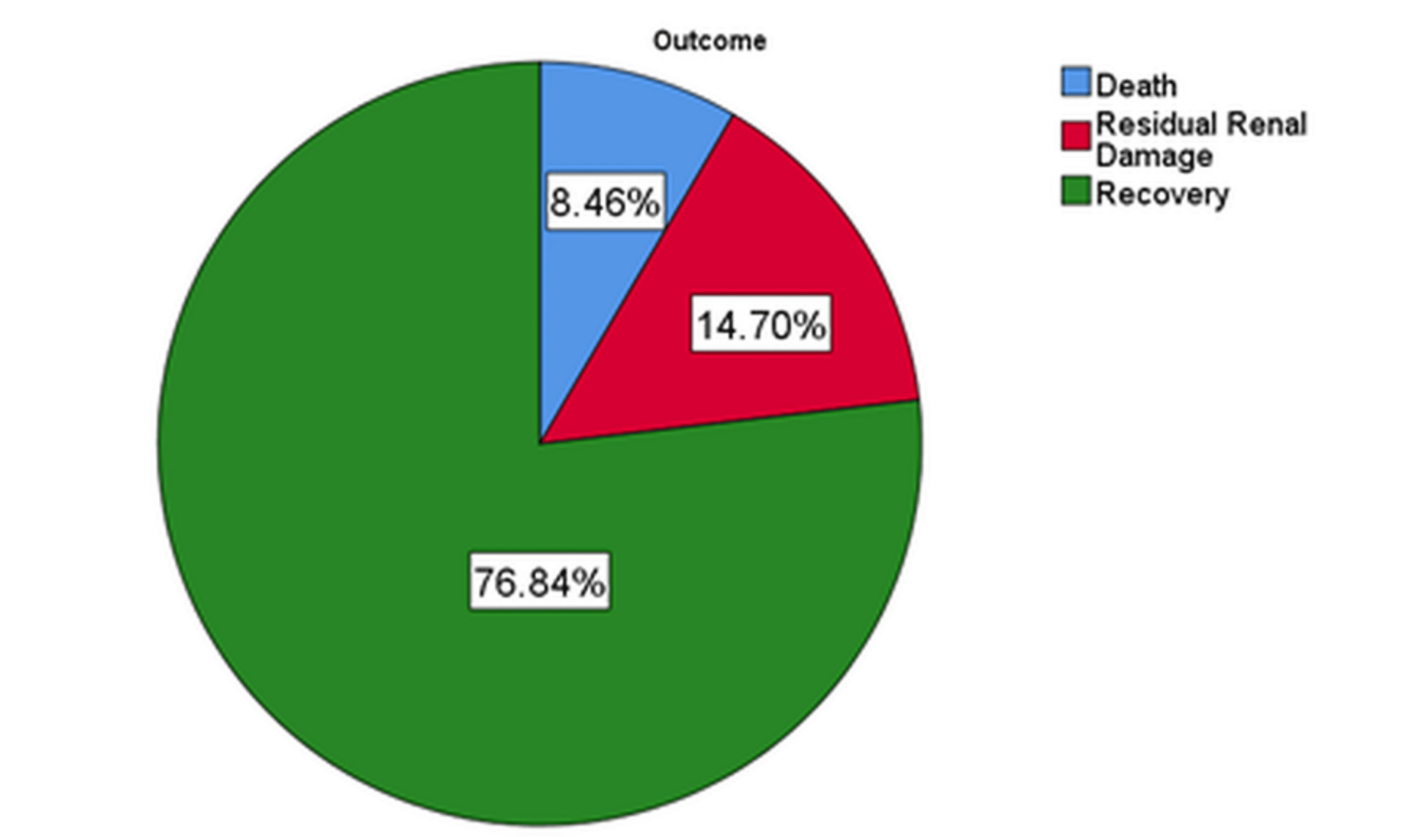
Outcomes of patients with acute kidney injury

Residual renal damage was found among 66 (14.7%) children who developed AKI. Another 38 (8.46%) patients passed away, suggesting a higher mortality rate in children who developed AKI than in those without AKI (Figure 7).

**Figure 8:**
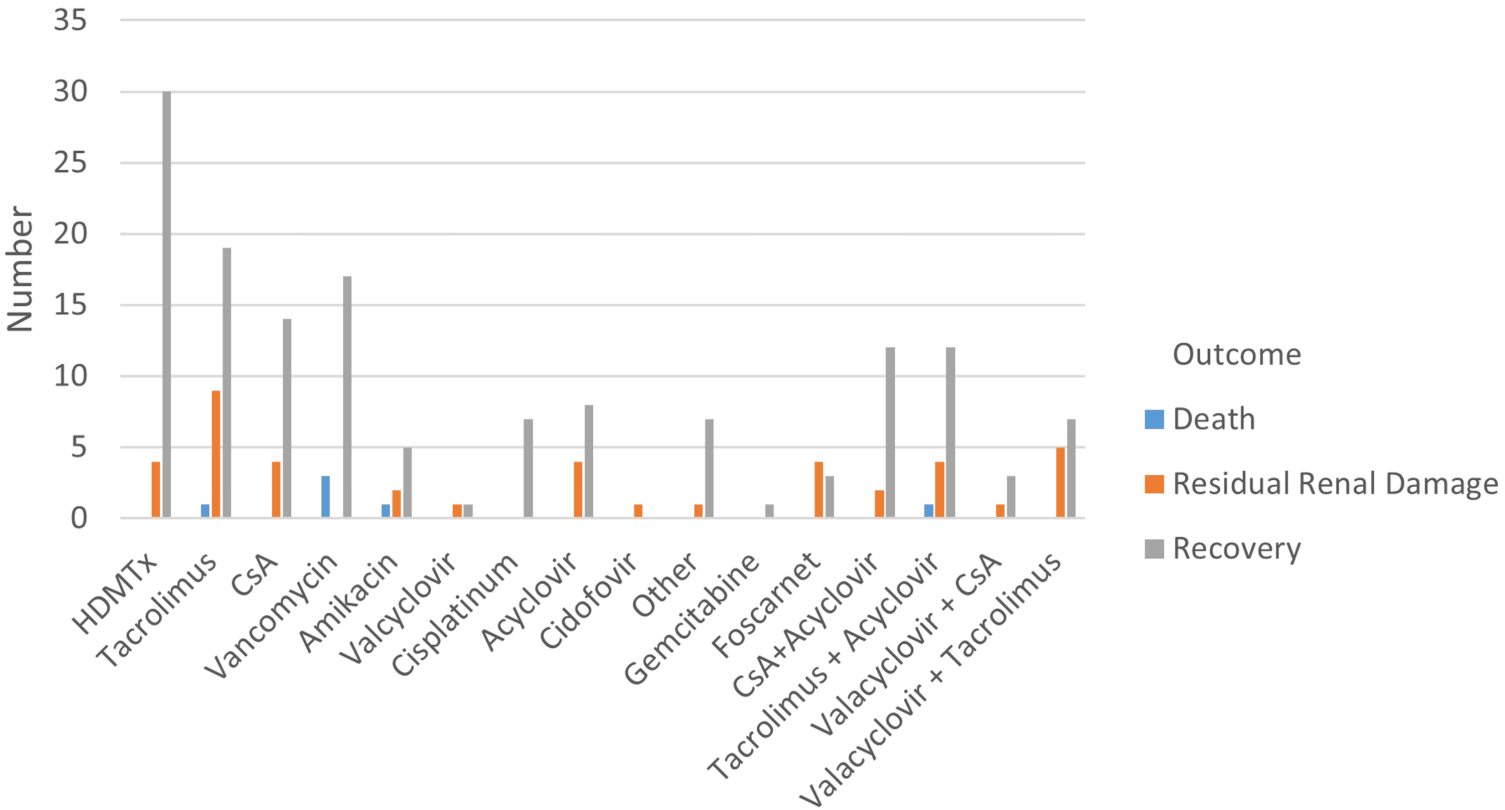
Outcomes associated with nephrotoxic medications HDMTx: high-dose methotrexate; CsA: cyclosporine A

Among children with AKI due to high-dose MTX, four (9.5%) developed residual renal damage. Foscarnet was strongly associated with residual renal damage, as five (57.1%) out of seven patients in this subgroup were affected. Relative risk analysis showed that foscarnet therapy was associated with a 2.7-fold higher risk of residual renal damage compared with non-foscarnet treatment, a statistically significant finding (P < 0.005). The most common drug causing residual renal damage at KHCC was tacrolimus, affecting nine (21.4%) patients of the total (Figure 8).

When comparing recovery and maximum stage of AKI reached, 59.4% of those with stage 3 AKI recovered complete renal function as compared to 83.4% of stage 2 and 80.6% of stage 1 (P < 0.001), whereas 23.8% of those in stage 3 maximum AKI passed away as compared with 7% in stage 2 and 1.6% of stage 1 (P < 0.001). The fact that 17.8% of children in stage 1 AKI ended up having residual renal damage indicates the importance of even minor elevations of serum creatinine (Table 4).

**Table 4:** Residual renal damage AKI: acute kidney injury

		Death	Damage	Recovery
AKI maximum	Stage 1	1.6%	17.8%	80.6%
	Stage 2	7.0%	9.6%	83.4%
	Stage 3	23.8%	16.8%	59.4%
Total		8.5%	14.7%	76.8%

Renal replacement therapy (RRT) was performed in 15 patients (3.2%), whereas 458 patients (96.8%) did not require RRT. The most common modality was hemodialysis, which was initiated in 12 patients, followed by CRRT in two patients and peritoneal dialysis in one patient (Table 5).

**Table 5:** Renal replacement therapy (RRT) CRRT: continuous renal replacement therapy

	Frequency	Percent (%)
No RRT	458	96.8
CRRT	2	0.4
Hemodialysis	12	2.5
Peritoneal Dialysis	1	0.2
Total	473	100.0

## Discussion

Despite the fact that there is increasing awareness of pediatric AKI in developing countries, data regarding AKI is still very scarce when compared with developed countries [16,7]. Different subgroups of patients have different characteristics of AKI. Data regarding oncology children is even more limited in developing countries.

Sutherland et al. [10] reported an incidence of 3.9/1000 admissions in a cross-sectional analysis of the 2009 Kids Inpatient Database; however, these were not oncology patients. Alsabbagh and Hashmat [17] reported an incidence among pediatric oncology patients in the United States of 18 per 1,000 admissions, which is very similar to the incidence we are reporting at KHCC.

A higher incidence among patients rather than admissions is anticipated. Xiong et al. [16] reported an incidence of AKI among Chinese children with cancer of 16.9%, of whom 5.6% were community-acquired and 11.3% were hospital-acquired, which is somewhat higher than the incidence we are reporting; however, the ratio of community-acquired AKI to hospital-acquired AKI is almost the same. The difference in incidence between patients and admissions is because oncology children often have multiple admissions for chemotherapy or complications related to chemotherapy and the original disease.

It is surprising that the most common location of developing AKI at KHCC, regardless of the cause, was in the home setting, accounting for almost one-third of cases. This raises a red flag as to why this is happening and what can be done about it; however, one should always remember that these are oncology patients and often have repeated vomiting and decreased oral intake due to chemotherapy as well as the original disease. They are also often discharged on nephrotoxic medications, which are taken for a long time at home. Still, this indicates that more support needs to be given to this group of patients in the home setting in order to prevent AKI.

The incidence of AKI differed across hospital settings, with the lowest rates observed in the general pediatric ward and the highest in the BMT unit and ICU. This pattern is not surprising, as children undergoing bone marrow transplantation or intensive care are typically exposed to more nephrotoxic medications and have greater illness severity. These findings suggest that routine monitoring and early nephrology involvement may be particularly important in high-risk settings such as the BMT unit and ICU, where the burden of AKI is greatest. Kaddourah et al. [5] reported an incidence of 26.9% in critically ill children admitted to the ICU, which is much higher than what we are reporting in our ICU; however, surgical patients postoperatively are usually admitted to the ICU at KHCC. This might explain the relatively low incidence in our ICU. As expected and reported elsewhere [5], stage 1 AKI was the most common among all patients and all locations [11].

Stage 1 AKI was more common in all locations at diagnosis and as the maximum stage reached, except in the ICU, where stage 3 (severe AKI) was the most common maximum stage reached. This is totally expected, as stage 3 AKI is more common in critically ill patients, as reported by Kaddourah et al. previously [5].

The changing etiology of AKI is now well documented, shifting from primary glomerular diseases to hospital-acquired AKI due to multiple nephrotoxins, post-transplantation, malignancy, post-surgical causes, and critically ill status [3,4]. It is well known that NTDs are a major cause of AKI nowadays [2,4]. Our study demonstrates that the most common cause of AKI in children with cancer at KHCC was exposure to nephrotoxic medications, followed by pre-renal AKI and TLS.

High-dose MTx nephrotoxicity, defined as higher than 500 mg/m^2^, is well documented in the literature, with AKI occurring in 2-12% of patients receiving high-dose MTx [15]. Nephrotoxicity results from crystallization of MTx in the renal tubular lumen, leading to tubular toxicity [18].

Lee et al. reported an incidence of AKI in children with hematological malignancy receiving acyclovir of 17.5%; it was noted that the combination with another NTD was a risk factor for AKI development [19]. At KHCC, the most common malignancy in children developing AKI was hematological malignancy, and it was noted that combining calcineurin inhibitors with antiviral agents was responsible for most of the AKI attacks in the BMT unit (80.6%). This supports what has been reported by Lee et al. and mandates that utmost care should be taken when combining acyclovir or valacyclovir with other nephrotoxic medications, especially calcineurin inhibitors.

The link between AKI and residual renal damage is now well established. Recent systematic reviews of the adult population showed a pooled rate of CKD after AKI ranging from 10.17 to 25.8 cases per 100 person-years [20,21]. At KHCC, 14.7% of children who had AKI developed CKD.

Amitai et al. [18] reported that AKI due to MTx toxicity is fully reversible; our data show that this is not necessarily true, as 11.8% of children with AKI due to high-dose MTx had residual renal damage. The fact that 17.8% of children in stage 1 AKI ended up having residual renal damage indicates the importance of even minor elevations of serum creatinine. The study has some limitations. First, it is a retrospective, single-center analysis derived from an institutional registry, which may limit the generalizability of the findings. Additionally, we were unable to perform multivariate analysis because most patients presented with multiple concurrent risk factors. In such cases, the most clinically plausible predominant risk factor was assigned as the primary attributed cause of AKI, which may introduce classification bias.

## Conclusions

AKI in children with cancer, despite similarities with other subgroups of children, has some important special features that are not present in other subgroups. The risk factors involved in AKI differ from one center to another, and this analysis further illustrates that there are differences in risk factors even in different locations within the same center. The study illustrates the importance of establishing an in-center, and possibly a national, registry for AKI, as this enables nephrologists to diagnose, trace, and maintain follow-up for children who develop AKI.
